# Community-based intervention improves abortion knowledge and reduces abortion stigma among women in Oromia, Ethiopia: a quasi-experimental mixed methods evaluation

**DOI:** 10.1186/s12905-022-02054-9

**Published:** 2022-11-21

**Authors:** Bekalu Mossie Chekol, Sally Dijkerman, Samuel Muluye, Dereje Wondimu

**Affiliations:** 1 Ipas Ethiopia, P.O. Box 63001, Bole Atlas Area, Addis Ababa, Ethiopia; 2 Ipas, P.O. Box 9990, Chapel Hill, NC 27515 USA

**Keywords:** Abortion stigma, Abortion knowledge, Abortion attitudes, SABAS, Difference-in-differences, Participatory evaluation, Quasi-experimental design, Ethiopia

## Abstract

**Background:**

Since liberalization of the Ethiopian abortion law, there have been significant improvements in the availability and utilization of facility-based abortion services in the country. However, nearly half of abortions still take place outside of health facilities, where the quality of procedures remains unknown. Abortion stigma is one reason that unsafe abortion persists. This study aims to evaluate the effect of community interventions conducted from 2016 to 2019 on the level and manifestation of abortion stigma and knowledge in a community in Oromia region, Ethiopia.

**Methods:**

The study is a quasi-experimental mixed methods evaluation including intervention and comparison communities. Two cross-sectional structured household surveys with independent samples, participatory evaluation wheels, and participatory impact diagrams were conducted with women of reproductive age (15–49) living in the communities. The baseline was conducted in 2016 and the endline in 2019. Difference-in-differences analysis was used to estimate the effect of the intervention on abortion knowledge and Stigmatizing Attitudes, Beliefs, and Actions Scale (SABAS) scores in the intervention community.

**Results:**

One thousand five hundred fifty-five women participated in the household survey and 28 women participated in participatory evaluation meetings. Over one-third (37%) of women surveyed in the intervention community were exposed to the intervention activities. Knowledge of one or more indications of legal abortion increased from 21 to 85% in the intervention community, compared to an increase from 30 to 57% in the comparison. Mean SABAS scores decreased by 9.3 points in the intervention community and increased by 5.3 points in the comparison community. Differences-in-differences models indicate that exposure to the intervention resulted in decreased stigma scores (coefficient = − 9.33, *p* < 0.001) and increased knowledge (coefficient = 0.26, *p* < 0.001).

**Conclusions:**

This is one of the first studies to measure changes in community-level abortion stigma and knowledge over time in Ethiopia using a mixed method, quasi-experimental design. The results indicate that the community-based intervention improved abortion knowledge and reduced abortion stigma.

## Plain English Summary

Despite abortion services being legal and widely available in Ethiopia, abortion stigma and limited knowledge of the law contribute to unsafe abortions still happening. Few studies in Ethiopia assess a community intervention’s ability to reduce abortion stigma and increase knowledge over time. We aimed to find out whether a community intervention implemented from 2016 to 2019 in Oromia region, Ethiopia was able to decrease stigma and increase knowledge regarding abortion. We measured stigma and knowledge among women ages 15–49 before and after the intervention, both in the intervention community and in a similar community with no intervention. We held community meetings and conducted surveys to gather qualitative and quantitative data. The results show that the intervention was successful at increasing knowledge and decreasing stigma. We identified which intervention components were most successful and can be used by others that have a similar goal. We recommend that future interventions include community member participation and leadership at all stages of the project; establish partnerships with local community groups and institutions; and conduct thorough testing of all communications materials with community members.

## Background

Nearly half of the estimated 55.7 million abortions occurring annually worldwide are considered unsafe. Almost all unsafe abortions (97%) happen in developing countries, resulting in 47,000 people dying and many more suffering injuries each year [1]. One such country, Ethiopia, has one of the most progressive abortion laws in Africa after a revision of the penal code by parliament in 2005, which expanded the legal indications for abortion. Since liberalization, efforts by the Ethiopian government and its partners have led to significant improvements in the availability and utilization of facility-based abortion services in the country. The percent of induced abortions being performed in health facilities increased from 27 to 53% from 2008 to 2014 [2]. In the same time period, the number of health facilities offering first trimester abortion care increased from 149 to 823 facilities, and those offering abortion care at or after 13 weeks similarly more than doubled from 29 to 66 by 2014 [3]. The number of people seeking treatment for postabortion complications nearly doubled, and an estimated 47% of abortions took place outside of health facilities [2]. The concurrent trends prompted questions about what factors beyond legal context and availability of services influence women’s abortion care-seeking, decision-making, and experiences in Ethiopia.

Research shows that one reason unsafe abortion persists, even in less restrictive legal environments such as Ethiopia, is the stigma surrounding abortion. Due to this stigma, women across countries and sociocultural contexts report undergoing social isolation, fear of social judgment, and psychological distress as part of their abortion experience [4]. Stigma occurs not only on the individual or interpersonal levels, but is manifested at all levels of society, including institutional, structural, and community levels [5–7]. Kumar, Hessini, and Mitchell conceptualized abortion stigma as a compound stigma “which builds on other forms of discrimination and structural injustices” [8] (p. 634), such as gender roles and inequalities. In line with researchers before them [5, 7], they argue that research on abortion stigma should focus on the community rather than individual or psychological levels.

Premarital sex, extra-marital pregnancy, and abortion are heavily stigmatized in many cultural contexts, and Ethiopia is no exception. Studies on abortion stigma in Ethiopia have largely focused on healthcare providers [9–14], some finding that favorable attitudes about abortion correlate with better knowledge of the law and experience providing abortion services [9, 10]. Previous research has quantitatively measured women’s attitudes about abortion in Amhara, Southern Nations, Nationalities, and People’s Region (SNNPR), and Oromia in Ethiopia [15–17]. A 2014 community survey conducted by Wado and colleagues found that the majority of women agreed that abortion among married and unmarried women is sinful or bad, concluding that abortion stigma may be a barrier to comprehensive abortion care [17]. These studies have advanced our knowledge of stigma in Ethiopian communities, but are limited by their cross-sectional, nonexperimental design.

Research on individuals’ experiences with stigma after having an abortion in Ethiopia is less common but does exist. Qualitative interviews conducted by Kebede et al. [18, 19] and Zenebe and Haukanes [20] provide insight into Ethiopian women’s experiences. Kebede and colleagues found that, when deciding where to access abortion services, young women who had an unsafe abortion considered social safety to be more important than medical safety [19]. They feared that disclosure of their abortion would lead to a significant and permanent decline in their social status. Interviews at Ethiopian universities by Zenebe and Haukanes revealed that students facing an unintended pregnancy often terminated their pregnancy secretly to avoid the stigma attached to premarital pregnancy, fearing shame and isolation from their families [20].

As long as stigma persists, improving the availability and accessibility of comprehensive abortion care in health facilities alone will not meet the needs of people seeking abortion. The primary objective of this study is to evaluate the effect of a community intervention on the level of abortion stigma and knowledge in Boset woreda (Ethiopian term for district) in Oromia region. We accomplish this by measuring changes in the level of stigmatizing attitudes, beliefs, and actions among community members in intervention and comparison communities before (2016) and after (2019) implementation of a community intervention by Ipas Ethiopia^1^ and a community-based organization (CBO), Oromia Development Association (ODA). This is the first study to apply the Stigmatizing Attitudes, Beliefs, and Actions Scale (SABAS) [21] among a general population of women of reproductive age in Ethiopia. The quasi-experimental design and application of community participatory evaluation methods strengthen this study’s relevance to stakeholders working in the field of sexual and reproductive health and offers direction for achieving similar results in other communities and countries.

### Interventions to improve abortion knowledge and reduce stigma

Ipas Ethiopia partnered with ODA to implement the community intervention in Boset woreda from July 2016 to February 2019. Baseline results informed the design and messaging used in community interventions to reduce abortion-related stigma as a barrier to safe abortion care. The intervention began by building the capacity of local community educators to identify and challenge stigmatizing language and use de-stigmatizing messages to educate the local community on safe abortion. Community educators included Health Extension Workers (HEWs), the frontline health workers for Ethiopia’s primary health care system, and Women’s Development Army (WDA) leaders and volunteers. A Women’s Development Army is a network of five to six women’s development teams which come together under one leader, each team including women from about five neighboring households. HEWs and WDA members participated in a series of orientations and trainings on sexual and reproductive health (SRH) and abortion, with a focus on abortion stigma.

Trainings included a discussion of the meaning of abortion stigma, its various domains, and how each domain manifests itself to impact community members’ access to safe abortion care. Values Clarification and Attitudes Transformation (VCAT) activities developed by Turner and Chapman Page [22] and adapted by Ipas Ethiopia for the local context were also conducted. The baseline survey and qualitative results not presented here [23] were used to craft the messaging and content of these trainings to ensure local relevance. This consisted of in-depth interviews with women who had an abortion and focus group discussions with school-aged girls about their knowledge and experiences with abortion stigma in Boset. Community educators were trained on communication skills, how to discuss sensitive issues including abortion, and how to effectively respond to the community’s questions regarding abortion.

Messages were developed and pretested with community members and shared with community educators. The core messages focused on unsafe abortion, maternal mortality, the intent of Ethiopia’s abortion law, the right to abortion, religion and abortion, misconceptions about abortion, and examples of specific actions individuals can take to challenge stigma. Guiding messages to encourage non-stigmatizing treatment of people who have had an abortion were developed to portray people from different walks of life. These key messages explained that unwanted pregnancy can occur due to many factors and is not always under the control of those who experience it. Society and government institutions have the responsibility to provide information and services to anyone who may need or want safe abortion. Decision-making around abortion is highly personal, contextual, and influenced by multiple factors. Finally, those accessing abortion services should not be condemned or treated differently to others. These messages were put in information, education, and communication (IEC) materials such as posters and flyers. The IEC materials were posted in areas where community members frequently gather, including health posts, health centers, kebele (Ethiopian term for village) and woreda administration offices, and other places in Boset. Two fictional stories, or vignettes, about women who had experienced abortion stigma were also developed to trigger discussions.

The community educators in turn led sensitization workshops, community dialogue sessions, and other interpersonal communication activities. Youth leaders, district-level government officials, community leaders, elders, and other community members were all invited to participate. Sensitization workshops included stigma focused VCAT activities, examples of specific actions individuals can take to challenge stigma, information about the abortion law and its intent, and where to seek safe abortion services. Interpersonal communication included community dialogue sessions and home-to-home visits, sometimes accompanied by traditional coffee ceremonies, using the IEC materials and vignettes to spark discussion. These activities reached more than 68,000 community members with stigma focused education, roughly 66% of Boset’s projected population over 15 years old in 2019 [24]. Each person generally received two exposures to activities, 2-4 weeks apart. Individuals encountered through these activities were provided with referrals and accompaniments to local health facilities for safe abortion services upon request. Throughout the intervention, project performance and activities were periodically reviewed with HEWs, abortion providers, health managers, and WDAs to ensure fidelity to the intervention design.

## Methods

### Study aim, design, and sample

We aimed to evaluate the effect of a community-based intervention on women’s knowledge, attitudes, beliefs, and actions regarding abortion in Oromia, Ethiopia. Although the community intervention targeted all community members, only women were included in the study sample for two main reasons: first, budget constraints prevented us from being able to obtain a representative sample of all community members; second, we theorized that women of reproductive age are impacted the most by abortion stigma in these communities and therefore would be more likely to witness changes in community-level abortion stigma. We employed a participatory, quasi-experimental mixed methods evaluation design including one intervention and one comparison community. Methods included two cross-sectional structured household surveys with independent samples, participatory evaluation wheels, and participatory impact diagrams (Table 1). The sample was women of reproductive age (WRA), defined as ages 15–49, living in the intervention and comparison woredas. The baseline was conducted in 2016 and the endline in 2019.

**Table 1:** Sample sizes by data collection method

Data Collection Method	Year	Sample Size (# of women of reproductive age)	
		Fogera (Comparison)	Boset (Intervention)
Cross-sectional structured household surveys with the SABA Scale	2016	349	351
	2019	430	425
Participatory evaluation meetings with participatory impact diagrams and evaluation wheels	2019	N/A	28

Two woredas were chosen purposively at baseline: Boset woreda in East Shewa Zone, Oromia region and Fogera woreda in South Gondar Zone, Amhara region. The criteria used to select these communities were intended to ensure that results were comparable. Woredas were selected from different regions to prevent the potential for intervention exposure to cross over into the comparison community. Each woreda had at least one health facility in the area where Ipas had an ongoing CAC intervention. Neither had a history abortion-focused community-level interventions in the area prior to Ipas and ODA’s work following the baseline. The communities were socio-demographically similar as of the most recent census in 2007. Both woredas were predominantly Orthodox Christian (with 96% of women in Fogera and 61% in Boset), followed by Muslim (4% of women in Fogera and 16% in Boset), and Protestant (< 1% of women in Fogera and 8% in Boset). Both were largely rural communities, with 89% and 81% living in rural areas in Fogera and Boset, respectively. Education rates among women were also similar; 75% of women in Fogera and 68% in Boset had never attended any formal schooling [25, 26]. After the baseline, Boset was chosen to be the intervention community due to the presence of a strong potential community partner (ODA), which was important to facilitate the intended intervention. Fogera served as the comparison community.

For each woreda, we used multi-stage cluster sampling to select a representative sample of WRA. As the primary sampling unit, three to four kebeles were selected from each woreda, seven total, from a list obtained from the two Woreda Health Offices. Within each woreda, kebeles were split into two categories: urban (population greater than 2000 people) and rural (population less than 2000 people). Since both woredas consisted of approximately 80% rural kebeles, we then randomly selected two to three rural kebeles and one urban kebele per woreda. Selected kebeles included Olenchitni (urban), Doni (rural), Qarwa Mirkessa (rural), and Tadacha (rural) from Boset and Alember (urban), Abuhakokit (rural), and Wotemb (rural) from Fogera.

The secondary sampling unit was the household level. For each selected kebele, we acquired a list of all households with WRA in each village from local HEWs. An updated list was acquired for endline sampling. Based on the relative WRA population size of each kebele, we selected a proportional number of households from each at baseline and endline. WRA residing in the selected households were recruited for participation in the study. When multiple eligible women lived in the same household, one was randomly selected.

We set a power of 80% and a 95% confidence level to minimize Type II and Type I errors, respectively, and a 10% response rate based on the high response rate achieved by Wado et al. in a similar study setting [17]. As the SABAS had not been measured longitudinally in an Ethiopian setting prior to this study and is closely tied to dominant social and gender norms that can take a long time to change, we set a conservative expected effect size on stigma score reduction of 8 percentage points to ensure a large enough sample to detect a modest expected effect size (details on the scale are provided under “Data Collection”). The target sample size was 427 women per woreda per wave of data collection, for a total of 854 women per wave and 1705 women overall. The actual response rates were 84% in 2016 and 88% in 2019. Survey respondents were not provided with any remuneration.

### Data collection

All study tools were designed in English, translated to Amharic and Oromo, and subsequently back translated to English. Tools were then reviewed by a team of native English, Amharic, and Oromo speakers for accuracy. We made additional modifications to the translated tools during data collector trainings and piloting to ensure high quality translation. A structured paper-based questionnaire was collected at baseline and endline in Boset and Fogera. The questionnaire asked about participant demographics, knowledge of abortion, and the SABAS [21] to quantitatively measure abortion stigma. We chose the SABAS as it was the only existing scale created to measure community-level abortion stigma validated in an African setting (specifically, Ghana and Zambia).

We used participatory evaluation methods to collect qualitative data in Boset at endline to assess intended and unintended changes in abortion attitudes and beliefs within the larger community. In each surveyed kebele, a facilitator skilled in participatory methods facilitated meetings with a goal of 25–40 community members who had been exposed to the intervention. Community-based partners that helped implement intervention recruited women ages 18 and older, but the partners themselves were not present at the meetings. At each meeting, participants completed an evaluation wheel and a participatory impact diagram, taking around 2-3 h total. Participants received 100 Ethiopian Birr in remuneration for their attendance at the meeting.

In the evaluation wheel activity, adapted from the International Fund for Agricultural Development (IFAD) [27], the facilitator asked participants to describe the attributes of the community intervention that would make its implementation and activities ideal. The ‘ideal state’ of the intervention was identified, with the responses portrayed on separate cards and arranged in a circle on the ground. Participants rated each of the attributes with small objects to illustrate how successful they believed the project to be in achieving each. For the participatory impact diagram activity, adapted from Kariuki and Njuki [28], participants drew the positive and negative results of the intervention in their community. Impact was defined as an individual or community wide experience, positive or negative, expected or unintended, resulting directly or indirectly from the intervention. Outputs from these methods included audio recordings, flip charts, photographs of flipcharts, and facilitator notes.

### Variables

Exposure to Ipas’s intervention was defined as the woman having attended one or more safe abortion events in her community (one or more events was categorized as attended event = 1 and no events = 0). The dosage of exposure to the intervention was defined as the number of times the woman attended safe abortion events in her community (categorical: 1 = Only once; 2 = twice; 3 = three or more times). The latter was only captured for women who reported attending at least one event at endline.

Two primary outcomes were collected in the survey. First, abortion stigma was measured using SABAS, an 18-item scale using Likert questions developed by Shellenberg and colleagues [21]. The scale contains three subscales: fear of contagion (3 items), negative stereotyping (8 items), and exclusion and discrimination (7 items). Certain items were reverse coded. Possible SABAS scores range from 18 to 90, higher scores indicating higher levels of stigmatizing attitudes, beliefs, and actions regarding abortion [21]. SABAS scores were analyzed both as mean total scores and in quartiles. Quartiles were created by splitting scores into categories (18–36; 37–53; 54–71, 72–90), with higher scores indicating higher stigma. The objective of SABAS quartiles is to see scores move from the higher two to the lower two categories, signifying a decrease in stigma.

The second outcome collected was knowledge of legal indications of abortion. Women were asked to list known legal indications of abortion in Ethiopia, which include pregnancy from rape/incest, risk to the fetus, risk to the pregnant person, the pregnant person is under age 18, and the pregnant person is physically or mentally disabled. This outcome was categorized as a binary variable, where being able to state one or more indication was categorized as having some knowledge (=1) and knowing zero indications was categorized as having no knowledge (=0) of legal indications.

### Data analysis

Survey data were entered using EpiInfo at baseline, CSPro 7.1 at endline, and both were transferred to a Stata file type using StatTransfer. Statistical analysis was conducted using Stata SE 16 and R. Sociodemographic characteristics included age, current schooling, highest level of school completed, marital status, and religion. Descriptive analysis included frequencies and percentages for categorical variables and mean, standard deviation, median, min, and max for all continuous variables. All variables were analyzed by wave (2016 = baseline; 2019 = endline) and intervention group (Boset = Intervention; Fogera = Comparison).

Bivariate analysis included Chi-Square tests of independence for categorical variables, Fisher’s exact test when expected cell counts were less than five, and Wilcoxon-Mann-Whitney tests for continuous variables (age and SABAS scores). Bivariate tests were performed for cross-woreda comparisons within each wave and cross-wave comparisons within each woreda. We tested reliability of the scale for the Ethiopian context by calculating Cronbach’s alpha of reliability, using a coefficient ≥ 0.60 as a cutoff for acceptable reliability [29]. Using the lavaan package in R [30], we conducted confirmatory factor analysis (CFA) of the 3-factor scale to test the SABAS’s validity and calculated the following goodness-of-fit statistics: root mean square error of approximation and standardized root mean squared residuals (RMSEA and SRMR, acceptable cutoffs of < 0.08) and the comparative fit index (CFI, acceptable cutoff of > 0.9).

We conducted difference-in-differences (DID) analysis for the stigma and knowledge outcomes separately. The DID variable in both models was defined as the interaction between intervention group (1 = Intervention; 0 = Comparison) and time (1 = Endline; 0 = Baseline). We used linear regression to conduct the first model using total SABAS score as the outcome. We used logistic regression and knowledge of legal indications of abortion as the outcome for the second model. For both outcomes, we first fit unadjusted models with the outcome and DID variable. We then analyzed covariates for potential confounders that may violate the common trends assumption that underlies DID analysis. We made model adjustments to address threats to DID assumptions in accordance with recommendations in statistical literature [31–33]. Specifically, using linear regression, Chi-square tests, and Wilcoxon-Mann-Whitney tests, we categorized all covariates according to their relationship to time (baseline vs. endline), treatment (intervention vs. comparison group), and effect on each outcome (SABAS score and knowledge). Covariates that had a time-varying effect on the outcome were considered confounders and adjusted for using an interaction variable between the covariate and time. Covariates that changed over time but had a time-invariant effect on the outcome were also considered confounders and were included as covariates in the model.

Products of the participatory evaluation methods were analyzed thematically to assess both intended and unintended changes in abortion-related attitudes and beliefs among the meeting participants. Audio recordings from participatory methods were transcribed verbatim. The transcripts and interview notes were coded by a research consultant using Microsoft Excel and NVivo software. After coding, matrices were created to aid descriptive analysis. Structural coding was applied based on prioritized topics and questions. The coded segments were organized in a matrix using Microsoft Excel and further coded for data reduction and analysis. Through this process, we identified major themes focusing on the impacts and implementation process of the project.

### Ethical approval

This study received ethical approval from the National Research Ethics Committee at the Federal Democratic Republic of Ethiopia Ministry of Science and Technology for the 2016 baseline and from the Oromia Regional Health Bureau and Amhara Region Public Health Institute Ethics Review Committees, respectively, for the 2019 endline. All study participants provided written informed consent.

## Results

### Results from household surveys

Seven hundred women at baseline and 855 women at endline participated in the household survey (*N* = 1555). Table 2 presents respondents’ demographic characteristics. Most respondents were over 24, with the mean age increasing more in Fogera over time (from 29 to 36 mean ages). Boset had more respondents under 25 years old in the sample at both time points (27% in Boset and 17% in Fogera in 2019). Higher levels of education were found in Boset, where only 26% of women had no formal education, compared to 81% of women in Fogera at endline. In both communities, secondary education and higher were rare. Marital status was similar across all samples, with over 80% of participants married. The greatest difference between the groups was religion. While women from Fogera were predominantly Orthodox Christian (93% in 2016), Boset included a greater variety of religious backgrounds, and only 39% were Orthodox Christian in 2016. The prevalence of Orthodox Christianity increased in both communities by 2019.

**Table 2:** Respondent demographics by woreda, 2016 and 2019

Respondent demographics	Fogera (Comparison)		Boset (Intervention)	
	2016 Baseline (***n*** = 349)	2019 Endline (***n*** = 430)	2016 Baseline (***n*** = 351)	2019 Endline (***n*** = 425)
	n (%)	n (%)	n (%)	n (%)
*Age (Mean, SD)*	29 (8)	36 (11)	28 (7)	29 (7)
≤ 18	12 (3)	12 (3)	12 (3)	10 (2)
19–24	91 (26)	62 (14)	114 (32)	108 (25)
≥ 25	237 (70)	344 (80)	222 (63)	307 (72)
Don’t know/refused	9 (3)	12 (3)	3 (1)	0 (0)
*Currently in school*				
Yes	23 (7)	29 (7)	14 (4)	44 (10)
No	325 (94)	400 (93)	330 (96)	381 (90)
*Highest level of schooling completed*				
No formal education	219 (63)	347 (81)	183 (52)	109 (26)
Primary school	72 (21)	46 (11)	113 (32)	162 (38)
Secondary school	41 (12)	28 (7)	44 (13)	71 (17)
Technical school	7 (2)	0 (0)	1 (< 1)	6 (1)
College or university	7 (2)	5 (1)	8 (2)	15 (4)
Don’t know/refused	1 (< 1)	1 (< 1)	2 (1)	62 (15)
*Marital Status*				
Married	274 (79)	367 (86)	312 (89)	356 (84)
Not married, steady partner	7 (2)	4 (1)	9 (3)	5 (1)
Single	35 (10)	25 (6)	9 (3)	29 (7)
Separated/divorced	21 (6)	15 (4)	8 (2)	23 (5)
Widowed	11 (3)	18 (4)	13 (4)	9 (2)
Refused	1 (< 1)	0 (0)	0 (0)	3 (1)
*Religion*				
Orthodox Christian	325 (93)	420 (98)	136 (39)	227 (53)
Protestant	1 (< 1)	0 (0)	68 (19)	102 (24)
Catholic	0 (0)	1 (< 1)	0 (0)	0 (0)
Muslim	21 (6)	8 (2)	136 (39)	90 (21)
Traditional	2 (1)	0 (0)	11 (3)	6 (1)
Other	0 (0)	1 (< 1)	0 (0)	0 (0)
No religious affiliation	0 (0)	0 (0)	0 (0)	0 (0)
Don’t know/refused	0 (0)	0 (0)	0 (0)	0 (0)

Table 3 shows respondents’ exposure to safe abortion events in their community. Despite Ipas having no community-level interventions in either community at baseline, women from both woredas reported having attended one or more safe abortion events in 2016, including 21% in Fogera and 19% in Boset. Aside from attending these events, some women reported receiving information about abortion from friends, family, and partners (*n* = 106), healthcare providers (*n* = 35), and HEWs (*n* = 32) (data not shown). By 2019, the intervention activities reached about one-third of the sample (37%) in Boset, a significant increase from baseline (*p* < 0.001). Among women in Boset who attended one or more events, coffee ceremonies (23%), school and university activities (28%), and women’s health development committee activities were most common (28%). Thirty-eight percent of women in Boset reported attending other community-based activities in 2019. Although it is not definitively known what these activities were, local program implementers believe this most likely includes home-to-home visits, peer education sessions, and other social gatherings led by the WDAs trained during the intervention. Among Boset women that attended any event, most had attended more than one (76%). Exposure to safe abortion information had decreased in Fogera by endline (6%), as no intervention activities took place.

**Table 3:** Exposure to safe abortion events by woreda, 2016 and 2019

Exposure to safe abortion events	Fogera (Comparison)		Boset (Intervention)	
	2016 Baseline (***n*** = 349)	2019 Endline (***n*** = 430)	2016 Baseline (***n*** = 351)	2019 Endline (***n*** = 425)
	n (%)	n (%)	n (%)	n (%)
*Attended one or more safe abortion events*	74 (21)	26 (6)	65 (19)	158 (37)
Coffee ceremonies	6 (8)	5 (19)	7 (11)	36 (23)
School/university activities	33 (45)	6 (23)	15 (23)	45 (28)
Women’s group activities	15 (20)	4 (15)	15 (23)	23 (15)
Youth group activities	12 (16)	6 (23)	7 (11)	11 (7)
Women health development committee activities	27 (36)	10 (38)	28 (43)	44 (28)
Other community-based activities	9 (12)	1 (4)	4 (6)	60 (38)
Other	2 (3)	0 (0)	5 (8)	0 (0)
*Number of times safe abortion events in community attended*^a^				
Only once	N/A	10 (38)	N/A	38 (24)
Twice		10 (38)		37 (23)
Three or more times		6 (23)		84 (53)

^a^Among those who attended at least one event. Question not asked at baseline

Results of the Cronbach’s alpha test of reliability for SABAS indicate acceptable reliability of the overall scale and its subscales. The full scale had an alpha of 0.91, and all subscales had an alpha above 0.60, signifying strong internal consistency. CFA results showed acceptable goodness-of-fit statistics for the 3-factor model, with an RMSEA of 0.071, SRMR of 0.051, and CFI of 0.096, all above the pre-determined acceptability cutoffs. Mean SABAS scores were similar at baseline (Fogera: 49.7; Boset: 48.7), indicating medium levels of abortion stigma (Table 4). By 2019, mean stigma scores had decreased by 9.3 points in Boset (to 39.4; *p* < 0.001). The percent of women with scores in the first quartile (18–36) increased from 13% at baseline to 45% at endline (*p* < 0.001). The following statements had the greatest decreases in the proportion of women who agreed or strongly agreed in Boset (data not shown): “a woman who has an abortion is committing a sin” (2016: 83%; 2019: 58%); “a woman who has an abortion brings shame to her family” (2016: 72%; 2019: 44%); “a woman who has had an abortion might encourage other women to get abortions” (2016: 45%; 2019: 22%); and “once a woman has one abortion, she will make it a habit” (2016: 39%; 2019: 19%). Meanwhile, the mean stigma score increased by 5.3 points in Fogera by 2019 (mean: 55; *p* < 0.001).

**Table 4:** Abortion knowledge and stigmatizing attitudes, beliefs, and actions by woreda, 2016 and 2019

Abortion knowledge and SABAS	Fogera (Comparison)		***p***-value^**1**^	Boset (Intervention)		***p***-value^**1**^
	2016 Baseline (***n*** = 349)	2019 Endline (***n*** = 430)		2016 Baseline (***n*** = 351)	2019 Endline (***n*** = 425)	
	n (%)	n (%)		n (%)	n (%)	
*Knowledge of one or more indications of legal abortion*	105 (30)	245 (57)	***	75 (21)	361 (85)	***
Pregnancy from rape/incest	52 (15)	142 (39)	***	58 (17)	253 (78)	***
Risk to fetus	55 (16)	135 (37)	***	26 (7)	282 (75)	***
Risk to mother	78 (22)	173 (48)	***	27 (8)	292 (78)	***
Mother is < 18 years old	36 (10)	31 (9)	NS	45 (13)	151 (40)	***
Mother is physically or mentally disabled	40 (11)	78 (22)	***	14 (4)	166 (44)	***
*Total SABAS Score*^*2*^						
Mean (SD)	49.7 (10)	55 (9)	***	48.7 (10)	39.4 (15)	***
Median	48	55		50	39	
(Min, Max)	(28, 74)	(32,81)		(22,70)	(18,78)	
*SABAS Score Quartiles*						
18–36	25 (8)	7 (2)	***	41 (13)	190 (45)	***
37–53	170 (56)	183 (43)		179 (55)	151 (36)	
54–71	108 (35)	224 (53)		105 (32)	79 (19)	
72–90	2 (1)	10 (2)		0 (0)	5 (1)	
Missing (1 or more questions missing from scale)	44	6		26	0	
*Negative Stereotyping SABAS Subscale*^*2,3*^						
Mean (SD)	25.7 (5)	27 (4)	***	25.2 (5)	20.2 (8)	***
Median	26	28		26	21	
(Min, Max)	(13,40)	(14,39)		(10,36)	(8,38)	
*Exclusion and Discrimination SABAS Subscale*^*2,4*^						
Mean (SD)	17.1 (5)	20.2 (5)	***	15.7 (5)	13.5 (5)	***
Median	16	20		15	13	
(Min, Max)	(8, 28)	(11, 32)		(7, 28)	(7, 30)	
*Fear of Contagion SABAS Subscale*^*2,5*^						
Mean (SD)	7 (2)	7.8 (2)	***	7.7 (2)	5.7 (3)	***
Median	8	8		8	5	
(Min, Max)	(3, 12)	(3, 14)		(3, 12)	(3, 14)	

^1^Result of the Wilcoxon-Mann-Whitney test (SABAS scores) or Chi-square test. Fisher’s exact used for expected cell counts < 5. * *p* < 0.05; ** *p* < 0.01; *** *p* < 0.001; NS = *p*-value is not significant at < 0.05 level

^2^Limited to those who answered all questions in the scale or subscale, respectively

^3^The Negative Stereotyping subscale contains 8 items, with possible score ranging from 8 to 40 points

^4^The Exclusion and Discrimination subscale contains 7 items, with possible score ranging from 7 to 35 points

^5^The Fear of Contagion subscale contains 3 items, with possible score ranging from 3 to 15 points

Knowledge of one or more legal indications of abortion was higher in Fogera than Boset in 2016 (Fogera: 30%; Boset: 21%). Sources of abortion information across both communities were similar and included friends and family members (53%), healthcare providers (42%), HEWs (39%), television (11%), and radio (10%) (data not shown). By 2019, both communities had significant increases in knowledge of legal indications for abortion (*p* < 0.001). Knowledge increased to a greater extent in Boset (Boset: 21 to 85%; Fogera: 30 to 57%). In Boset, knowledge of legal indications was significantly higher among women that attended a safe abortion event (91% vs. 65%, *p* < 0.001, data not shown). While knowledge increased with attendance of safe abortion events, it did not increase significantly with more exposures.

Table 5 presents adjusted DID models for abortion stigma and knowledge. Model 1 (outcome = SABAS score) indicates that living in the intervention community (the DID variable) decreased stigma scores by over nine points (coefficient = − 9.33, *p* < 0.001). Time-varying covariates with time-varying effects on stigma scores were included as interaction terms with time in the model, including any school (coefficient = − 4.59, *p* < 0.001); being Orthodox Christian (coefficient = − 2.01, not significant), and knowledge of legal indications of abortion (coefficient = − 3.46, *p* = 0.009). Knowledge was included in the adjusted model because of its theoretical mediating effects on abortion stigma. All interaction terms were associated with lower stigma scores. Attending any safe abortion events was adjusted for as a time-varying covariate with time-invariant effects on the outcome (coefficient = 1.53, *p* = 0.04).

**Table 5:** Difference-in-differences models for SABAS scores and abortion knowledge

Variable	Coefficient	Std. Error	95% Confidence Interval		***p***-value
**Model 1: Outcome = Total SABAS Score**					
DID (Treatment*Time)^a^	−9.33	1.49	−12.25	−6.42	< 0.001
*Adjustment Covariates*					
Treatment (Intervention = 1)	1.40	1.06	− 0.67	3.48	0.185
Time (Endline = 1)	10.04	1.77	6.58	13.51	< 0.001
Any school	−5.04	0.89	−6.78	−3.29	< 0.001
Any school*Time^b^	−4.59	1.26	−7.05	−2.12	< 0.001
Orthodox Christian	3.99	1.09	1.84	6.14	< 0.001
Orthodox Christian*Time^b^	−2.01	1.48	−4.92	0.89	0.175
Knowledge of any indications of legal abortion	−3.23	1.02	−5.23	−1.23	0.002
Knowledge of any indications of legal abortion*Time^b^	−3.46	1.32	−6.05	−0.86	0.009
Attended any safe abortion events^c^	1.53	0.75	0.07	2.99	0.040
**Model 2: Outcome = Knowledge of one or more indications of legal abortion**					
DID (Treatment*Time)^a^	0.26	0.06	0.15	0.37	< 0.001
*Adjustment Covariates*					
Treatment (Intervention = 1)	−0.14	0.04	−0.22	− 0.06	< 0.001
Time (Endline = 1)	0.32	0.06	0.19	0.44	< 0.001
Any school	0.15	0.03	0.09	0.21	< 0.001
Any school*Time^c^	−0.02	0.05	−0.11	0.07	0.672
Orthodox Christian	−0.08	0.04	−0.16	0.002	0.056
Orthodox Christian*Time^b^	0.02	0.06	−0.09	0.13	0.764
Attended any safe abortion events^c^	0.21	0.03	0.15	0.26	< 0.001

^a^DID is the difference-in-differences variable, an interaction term of treatment group (Boset vs. Fogera) and time (baseline vs. endline); 1 = intervention group at endline

^b^Time-varying covariates with time-varying effects on the outcome are adjusted for using an interaction term between the covariate and time

^c^Time-varying covariates with time-invariant effects on the outcome are adjusted for by including the term in the model

Model 2 (outcome = knowledge of legal indications) shows an increase in women’s knowledge of legal indications for abortion in Boset by endline. Women in Boset had 26% higher knowledge as a result of living in the intervention community (DID variable coefficient = 0.26, *p* < 0.001). Being Orthodox Christian (coefficient = 0.02, not significant) and having attended any school (coefficient = − 0.02, not significant) were both adjusted for using an interaction term with time, similar to Model 1. Attendance at any abortion events was adjusted for as a time-varying covariate with a time-invariant effect on knowledge, and it was associated with 21% higher knowledge in Boset at endline (coefficient = 0.21, *p* < 0.001).

### Results from participatory evaluation

Twenty-eight women ranging from 16 to 40 years in age (mean age of 25.5) attended one of three participatory evaluation meetings in Tadecha and Olenchiti villages in Boset. Participants identified several strengths of the intervention design and implementation during the evaluation wheel activities. They felt that the intervention effectively taught community members about SRH issues, particularly because the community educators were selected from local community groups and had experience in teaching family planning. Community members, leaders, and WDAs reported being consulted and involved during the initial stage of the project planning and design. The following quote from a participant explains:

*“We have been informed about the intervention from the very beginning as we are women development army leaders. Oromia Development Association (ODA) had consulted us at the initiation of the project and we were involved in project planning, planning was conducted in collaboration with other community members. Community leaders and other members of Women Development Army were also engaged during the training conducted at the start of and during implementation of the project and have been creating SRH awareness among community members.”* (Participant from community meeting in Olenchiti)

Following their training, the WDA leaders and HEWs began outreach activities teaching mothers and other community members about family planning, unplanned pregnancy, and safe and legal abortion, using the core messages they helped co-design to spark discussion and public discourse about the negative stereotyping of people who have had an abortion. Participants explained that community groups and leaders participated in trainings led by the community educators and supported awareness creation for those sessions. Educational aids were provided to health posts to teach community members about contraceptives and safe abortion.

The ranking component of the evaluation wheel helped identify areas where the intervention could be improved. Some participants felt that the trainings were not comprehensive or tailored to the specific community context enough. Additionally, some felt that not all community educators were considered knowledgeable professionals on the topics covered. As a result, participants recommended improving the training that implementers provided to community educators, particularly regarding communication and social skills. Somewhat related to this, participants recommended that the intervention activities be tailored to focus more on young girls ages 15–30 and should include more youth-friendly trainings: “*educators need to be trained more on communication and social skills to talk to adolescent and young girls comfortably and friendly”* (Participant from community meeting in Olenchiti). Community members also expressed a desire for stronger referral systems between the community and local health care services to improve accessibility and utilization of SRH services, as well as improved quality of care at local health facilities. Local health providers were considered by some to be inadequate in their interpersonal communication skills and respectful care for women seeking abortion.

In the participatory impact diagrams, women drew the outcomes of the intervention in their community, capturing both intended and unintended outcomes. Participants reported that the educational outreach conducted by the trained WDA leaders and HEWs led to increased awareness of family planning and safe abortion services among women and young girls. They agreed that more community members now know the benefits of family planning use and where to seek safe abortion in case of unwanted pregnancy. One participant explained:

*“Now the community understands where to seek for FP [family planning] and abortion services. Young girls and students started visiting health facilities in case of unintended pregnancy and for getting FP service. They start to discuss SRH issue with community members, educators and consult their issues with health workers to get FP and safe abortion services. This enables them to continue their education without interruption caused by unintended pregnancy”.* (Participant from community meeting in Olenchiti)

There was agreement that the intervention resulted in increased understanding of Ethiopia’s abortion law and, as a result, women’s confidence to seek safe and legal abortion.

Because of the continuous education and communication in the intervention, participants reported that fear of abortion stigma among women and girls had reduced over time. Before the intervention, lack of awareness, culture, and religious beliefs greatly influenced utilization of safe abortion and family planning services among community members. Following the intervention, women and girls better understood the risks of having unwanted pregnancy and using traditional and untrained providers for pregnancy termination.

*“As we all know women were not revealing unintended pregnancies to their husband, families and friends and resort to unsafe abortion in fear of community stigma. Today, things are improved, we started openly discussing about family planning and safe abortion services without fear.”* (Participant from community meeting in Tadecha)

*“I am a member of community care coalition. I remember an orphan student was raped and encountered unplanned pregnancy and in consultation with women and children office, we referred her to the nearby health facility where safe abortion service is provided, and she got the service and become free from unintended pregnancy burden. As a result, she continued her education without interruption.”* (Participant from community meeting in Tadecha)

Participants reported an increase in the number of community members who sought safe abortion care and contraception at health facilities, particularly long acting and reversible methods of contraception (LARC).

A notable, negative unintended outcome of the intervention was also reported. Community educators were sometimes labelled with negative identifiers and names by local community members: “*There are some individuals who consider educators as sinners for informing safe abortion services and rights to the community members. They give unnecessary or unpleasant names or labels to educators” (Participant from community meeting in Tadecha)*. Community leaders’ and heads of households’ engagement in abortion and contraception education did not live up to the participants’ desired level. Finally, although significant improvements have been observed over time, participants reported that in some areas of the community, many still have poor attitudes toward safe abortion.

## Discussion

Even after a multi-year community-based intervention, abortion stigma persists in Boset, albeit to a lesser extent. It cannot be ignored that abortion stigma increased in Fogera, despite encouraging knowledge gains. Negative stereotyping was the most pervasive form of stigma in both settings. We found that prior to any interventions, women in both communities had some knowledge of legal indications for abortion, and greater knowledge at endline was associated with exposure to intervention activities. Our analysis indicates that the community intervention implemented by ODA and Ipas successfully contributed to reducing abortion stigma and increasing knowledge of the legal indications for abortion among women in Boset, Oromia.

Prior to the intervention, less than one-third of women surveyed had knowledge of one or more legal indications for abortion (30% in Fogera; 21% in Boset). These findings suggest lower levels of knowledge than found in other communities. Bantie et al. [16] found that 43.3% women in Bahir Dar city in northwest Ethiopia had good knowledge of the law; Muzeyen and colleagues [15] similarly found that 42.8% of women in Bahir Dar had fair to good knowledge about legal indications. These differences may be due to the different geographic locations of the samples. Furthermore, the present study asked about the legal indications in an open-ended manner (i.e., “Under what conditions is abortion legal in Ethiopia?”), whereas the referenced studies asked about each indication separately, prompting women to answer if the indication is included in the law using the answer options “Yes,” “No”, or “Don’t know” [15, 16].

Despite the lack of any known community interventions in either community prior to Ipas’s intervention, about one-fifth of women in both Fogera and Boset reported attending a safe abortion event in their community at baseline. Clearly, safe abortion information was already being made available to these communities in some form. This is likely due to the presence of Ipas Ethiopia’s interventions support to local public health centers to provide comprehensive abortion care. Research in Bahir Dar similarly found that a primary source of abortion information for women in Amhara was healthcare providers, second to TV and radio [15]. While that study was conducted in the capital of Amhara, it suggests that women in our Fogera sample may have been exposed to information through TV or radio events originating in Bahir Dar city.

Abortion stigma was prevalent in both communities at baseline, with about a third of women scoring in the higher two SABAS score quartiles (54–90 points, indicating higher stigma), indicating high stigma levels. However, there was a large standard deviation of 10 points. Relative to exclusion and discrimination and fear of contagion, the negative stereotyping subscale had the highest levels of stigma in both communities. From 20% to 25% of women (Fogera and Boset, respectively) agreed that “I would try to disgrace a woman in my community if I found out she’d had an abortion” in 2016. Wado and colleagues’ 2014 survey in Oromia found slightly lower agreement with this statement, at 18% of women surveyed [17]. Similarly, the majority of women in our sample agreed that “a woman who has an abortion is committing a sin” (66% in Fogera, 83% in Boset), consistent with Wado and colleagues’ findings (69% and 79% of women agreed with this statement in regards to unmarried and married women, respectively) [17].^2^ Bantie et al. [16] similarly found that only 38% of women in Bahir Dar city, Amhara had a favorable attitude toward the abortion law. These results echo stories collected from young women, who report fear of shame and isolation [20] and would prefer to risk medical safety over social safety when seeking abortion care [19].

The community-based intervention was successful in reaching a substantial segment of the local population of women of reproductive age in Boset, Oromia. After two and a half years, over one-third (37%) of women had been exposed to intervention activities, with three-quarters of those women (76%) receiving multiple exposures. Women’s group and WDA activities reached three times as many women as youth group activities, consistent with participatory feedback that the intervention was less successful at reaching adolescent women. Both communities showed improvements in knowledge of legal indications for abortion. Knowledge was positively associated with attendance at a safe abortion community event. The adjusted difference-in-differences models reveal that the intervention community of Boset had greater increases in knowledge than the comparison community, Fogera.

The DID analysis also provides evidence that the intervention led to a reduction in abortion stigma in Boset. SABAS scores decreased by approximately 8 points in Boset after model adjustments. Knowledge of legal indications for abortion was associated with lower stigma scores. Participatory results were generally positive about a reduction in stigma, documenting stories that community members were more comfortable to seek abortion services and care-seeking at local public health centers increased during the intervention. Notably, participatory and SABAS results also highlight that stigma persists in both communities. For instance, the fact that some community educators involved in the intervention were labeled as “sinners” supports the SABAS findings that negative stereotyping is the most common form of abortion stigma in these communities, even following the intervention.

Lessons from this study should be applied to other community-based interventions in Ethiopia and beyond. We recommend that interventions in Ethiopian communities give special attention to negative stereotyping, the SABAS subscale with the highest levels of stigma. Community members identified several intervention components as vital to its successes. Ipas Ethiopia’s partnership with the Oromia Development Association was critical. Throughout the design, implementation, and evaluation of the intervention, the organizations provided structured space and time for meaningful community participation. Community members were designers, implementers, and evaluators of this work. As a result, the intervention activities drew on local institutions, such HEWs and WDAs, traditions such coffee ceremonies, and experienced local educators to bolster interpersonal communications. Other successful approaches we identified include using baseline mixed methods data to design intervention activities and messaging; testing all IEC materials and communications products with beneficiaries (e.g., the vignettes); and the training of and subsequent outreach by WDA leaders and HEWs to facilitate VCAT activities and generate public discourse about the negative stereotyping of people who have abortion.

Areas identified as priorities for future interventions include an increased focus on referral systems to the local health facilities (e.g., including providers in community events); improving reach of young people by developing youth-specific activities and meaningful youth participation at the design and implementation phases; and more comprehensive training for community educators with a focus on communicating about sensitive subjects. Community-delivered interventions should integrate psychosocial support opportunities for community educators, as they may experience stigmatization due to their activities providing abortion information.

This study has limitations that must be acknowledged. A strict assumption of difference-in-differences analysis is the common trends assumption. We found that religion and education were confounding variables with time-varying effects on stigma and knowledge. While it is impossible to know for certain whether these variables violated this assumption, we attempted to address them, nonetheless. As previously discussed, we undertook statistical procedures to adjust for this in the final models using interaction terms and explored other methods for determining the impact of these variables based on recommendations from other researchers [31–33]. Another limitation is the cross-sectional independent samples, which means that the study did not capture changes in outcomes at an individual level. Surveying the same sample of women at each wave would improve the rigor of future evaluations. This approach also resulted in significant differences in religion and educational status across samples within the same communities, limiting the comparability of the two samples over time. Although the two communities were socio-demographically similar per the 2007 census, the 2016 baseline showed significant differences in the expected religion and educational status of Boset women; this could be due to sampling bias brought on by the cluster sample, or it could be that the community experienced significant changes between 2007 and the 2016 baseline. Although the reason cannot be fully determined due to the lack of more recent socio-demographic data, this finding limits the comparability of Boset to Fogera. Last, results from the participatory evaluation activities are limited by the recruitment method. Because recruitment was conducted by community-based partners participating in the intervention, selected participants may have had reasons to give positive answers during the activities.

This study has several strengths. It is the first mixed methods, quasi-experimental study to measure changes in community-level abortion stigma and knowledge over time in Ethiopia. The intervention components highlighted in this paper can be repeated in similar settings to improve abortion knowledge and reduce abortion stigma in communities. Our evaluation of the psychometric properties of the SABAS also supports validity and reliability of this scale for use among women of reproductive age in Ethiopia.

## Conclusions

This evaluation provides evidence of promising strategies for improving abortion knowledge and reducing stigma in Ethiopian communities, yet abortion stigma persists despite the intervention’s successes. Continued focus of these organizations and/or other stakeholders is recommended to sustain and build upon these improvements in Boset, and scale-up to include other Ethiopian communities, including Fogera, is strongly recommended to ensure all people who may need abortion care in Ethiopia are able to access abortion care without fear of stigma. Future interventions should be mindful of the pervasive negative stereotyping of people accessing abortion and those providing abortion information and set up support systems for participating community members. Increased attention to community-health system referral systems and increasing meaningful youth participation at the design and implementation phases are also recommended strategies. Finally, we encourage researchers to continue testing and applying the SABAS among WRA in Ethiopia.

## Data Availability

The datasets used during the current study are available from the corresponding author on reasonable request.

## Abbreviations

SNNPR: Southern Nations, Nationalities, and People’s Region
INGO: International non-governmental organization
FMOH: Federal Ministry of Health
CAC: Comprehensive abortion care
CBO: Community-based organization
ODA: Oromia Development Association
SABAS: Stigmatizing Attitudes, Beliefs and Actions Scale
HEWs: Health Extension Workers
WDA: Women’s Development Army
SRH: Sexual and reproductive health
VCAT: Values Clarification and Attitudes Transformation
IEC: Information, education, and communication
WRA: Women of reproductive age
IFAD: International Fund for Agricultural Development
CFA: Confirmatory factor analysis
RMSEA: Root mean square error of approximation
SRMR: Standardized root mean squared residuals
CFI: Comparative fit index
DID: Difference-in-differences
FP: Family planning

## Footnotes

1 Ipas is an international non-governmental organization (INGO) working in partnership with the Ethiopian Federal Ministry of Health (FMOH) to reduce maternal morbidity and mortality from abortion by increasing access to comprehensive abortion care (CAC) and information.

2 The 2014 study by Wado and colleagues [17] used several statements from the SABAS, but did not collect the entire scale. Therefore, comparisons to the present study are limited to individual statements.
